# Comparison of CT and integrated PET-CT based radiation therapy planning in patients with malignant pleural mesothelioma

**DOI:** 10.1186/1748-717X-4-35

**Published:** 2009-09-16

**Authors:** Berrin Pehlivan, Erkan Topkan, Cem Onal, Gul Nihal Nursal, Oznur Yuksel, Yemliha Dolek, Melek Nur Yavuz, Ali Aydin Yavuz

**Affiliations:** 1 Department of Radiation Oncology, Baskent University Medical Faculty, Adana Medical and Research Center, Kisla Campus, Adana, Turkey; 2 Department of Nuclear Medicine, Baskent University Medical Faculty, Adana Medical and Research Center, Kisla Campus, Adana, Turkey

## Abstract

**Background:**

When combined with adequate tumoricidal doses, accurate target volume delineation remains to be the one of the most important predictive factors for radiotherapy (RT) success in locally advanced or medically inoperable malignant pleural mesothelioma (MPM) patients. Recently, 18-fluorodeoxyglucose positron emission tomography (PET) has demonstrated significant improvements in diagnosis and accurate staging of MPM. However, role of additional PET data has not been studied in RT planning (RTP) of patients with inoperable MPM or in those who refuse surgery. Therefore, we planned to compare CT with co-registered PET-CT as the basis for delineating target volumes in these patients group.

**Methods:**

Retrospectively, the CT and co-registered PET-CT data of 13 patients with histologically proven MPM were utilized to delineate target volumes separately. For each patient, target volumes (gross tumor volume [GTV], clinical target volume [CTV], and planning target volume [PTV]) were defined using the CT and PET-CT fusion data sets. The PTV was measured in two ways: PTV1 was CTV plus a 1-cm margin, and PTV2 was GTV plus a 1-cm margin. We analyzed differences in target volumes.

**Results:**

In 12 of 13 patients, compared to CT-based delineation, PET-CT-based delineation resulted in a statistically significant decrease in the mean GTV, CTV, PTV1, and PTV2. In these 12 patients, mean GTV decreased by 47.1% ± 28.4%, mean CTV decreased by 38.7% ± 24.7%, mean PTV1 decreased by 31.1% ± 23.1%, and mean PTV2 decreased by 40.0% ± 24.0%. In 4 of 13 patients, hilar lymph nodes were identified by PET-CT that was not identified by CT alone, changing the nodal status of tumor staging in those patients.

**Conclusion:**

This study demonstrated the usefulness of PET-CT-based target volume delineation in patients with MPM. Co-registration of PET and CT information reduces the likelihood of geographic misses, and additionally, significant reductions observed in target volumes may potentially allow escalation of RT dose beyond conventional limits potential clinical benefits in tumor control rates, which needs to be tested in future studies.

## Background

Malignant pleural mesothelioma (MPM) is a relatively rare but highly aggressive tumor with expected median survival of only 9 to 17 months [1,2]. Although currently it appears to be a rare tumor, its incidence is increasing throughout most of the world including Turkey, where it is epidemic in three villages of the Cappadocia region. Also, familial forms with autosomal dominant inheritance have been reported in this region [3,4].

Although there is no universally accepted standard treatment for MPM, currently the EPP is the most widely preferred treatment modality. However, due to significant procedure related modality and mortality 85 to 90% of patients are not eligible for this aggressive procedure [5,6]. In this context, radiation therapy (RT) as the sole treatment in the presence or absence of concurrent chemotherapy may be a good alternative in suitable patient population. However, RT planning (RTP) for MPM is difficult due to the large, irregularly shaped area at risk, the high doses required for local control, and the proximity of many radiosensitive structures such as the liver, ipsilateral kidney, heart, spinal cord, esophagus, contralateral lung, and the ipsilateral lung itself in inoperable cases. In the latter setting, which is a therapeutic challenge, the recent, more sophisticated RT techniques, including intensity-modulated radiotherapy (IMRT), image guided radiotherapy (IGRT), and especially helical tomotherapy (HT), are promising. However, similar with all other tumor sites, accurate target delineation is crucial when RT is considered as the sole treatment or as a component of oncologic treatment, and additionally when combined with adequate tumoricidal doses, accurate target volume delineation remains to be the one of the most important predictive factors for RT success in MPM.

Computed tomography (CT) is the primary imaging modality used in staging and RT planning for MPM. Rind-like extension of the tumor on the pleural surfaces is the most common CT feature [7]. However, CT often fails to accurately demonstrate transdiaphragmatic invasion and mediastinal lymph nodes [8,9]. Recently, 18-fluorodeoxyglucose positron emission tomography (PET) has demonstrated significant improvements in diagnosis, accurate staging, RTP, and assessment of tumor response to the prescribed treatment in a variety of tumor sites including the MPM [10-16]. PET imaging is based on biochemical processes that may offer better detection of tumors even before they become anatomically apparent. Integration of functional PET data with the detailed anatomical information of CT (PET-CT) has markedly increased the sensitivity, specificity and accuracy of discrimination between benign and malignant diseases, determination of tumoral extensions in to the mediastinum, abdominal cavity or pleural surfaces, medistinal lymph nodes or distant viscera [10,17,18]. In this context, integrated PET-CT provides more information, compared with ametabolic CT or nonanatomic PET. The high sensitivity and specificity of PET-CT in patients with MPM have been well documented. Benard et al analyzed 28 patients with suspected MPM and reported that specificity of PET was 100% with sensitivity of 91% in differentiating benign and malignant lesions [10]. Caretta et al found similar results, with accuracy of PET at 92% in the differential diagnosis of pleural diseases [17]. Similarly, in their recent report Plathow et al analyzed 54 patients with stage II and III MPM, and the authors reported accuracy of 77% for CT, 86% for PET, 80% for Magnetic Resonance Imaging (MRI), and 100% for PET-CT in patients with stage II disease, and accuracy of 75% for CT, 83% for PET, 90% for MRI, and 100% for PET-CT in patients with stage III disease [18].

To our knowledge, no studies address the role of PET-CT-based RTP in patients with medically inoperable MPM or in those who refuse surgery. Largely based on the aforementioned data, we hypothesized that using PET-CT data rather than CT data alone would change RT fields and possibly result in fewer geographic misses for unresected MPM. Therefore, in the present study, we compared CT-based and integrated PET-CT-based gross tumor volume (GTV) delineation and its subsequent expansion to clinical target volume (CTV) and planning target volume (PTV).

## Methods

Thirteen patients with histological diagnosis of MPM who were not candidate for a curative resection due to medical reasons or self refusal those who were treated with thorasic irradiation with a palliative intent are planned to be reassessed whether the intented target volumes may have changed if additional PET data was used in conjunction with CT compared to CT alone. This study was largely based on recent impressive high sensitivity and specifity data of PET in MPM diagnosis and staging as mentioned previously [10,17,18]. This pure delineation study protocol to evaluate the potential differences via implementation of PET-CT on palliative MPM cases was approved by the institutional ethic committee. Patients' charts were reviewed for the search of characteristics with nonmetastatic mesothelioma classified as T2-4 and/or N0-3 according to the International Mesothelioma Interest Group staging system [19], and no previous surgical resection.

As we acknowledged from patients' hospital records, each patient was placed in the supine position with both arms raised above their heads in a manner identical to treatment positioning during PET-CT. The PET-CT scan was performed in an integrated PET-CT system (Discovery-STE 8, General Electric System, Milwaukee, WI, USA). Patients were advised to fast for at least 6 hours before the PET appointment. After 370 to 555 MBq (10-15 mCi) 18-fluorodeoxyglucose was injected, patients rested for approximately 60 minutes in a comfortable chair. Preinjection blood glucose levels were measured to ensure that they were below 150 mmol/L. The patients were scanned on a flat-panel carbon fiber composite table insert. An enhanced CT scan from the base of the skull to the inferior border of the pelvis was acquired with 5-mm slice thickness, using a standardized protocol with 140 kV and 80 MA with contrast injection. The subsequent PET scan was acquired from the base of the skull to the inferior border of the pelvis as in the CT scan, using multiple-bed position. Attenuation was corrected by using the CT images. The processed images were displayed in coronal, transverse, and saggital planes.

After image acquisition, PET-CT data sets were transferred to our treatment planning system, Eclipse 7.5 (Varian Medical Systems, Palo Alto, CA, USA) into DICOM RT format, and the available data was utilized for planning purposes following image fusion. The CT-based and PET-CT-based treatment planning was computed for each patient. The target volumes were defined by the radiation oncologist (BP and checked by ET) with specific experience in MPM cancer treatment on the CT and integrated PET-CT images. The GTV was defined as the volume of macroscopic primary tumor and involved hilar and mediastinal lymph nodes identified on the planning CT. The CTV was created automatically with a 1-cm margin around the GTV with respecting to the natural anatomical barriers, such as vertebral column. The PTV1 encompassed the CTV plus a mean 1-cm margin, and PTV2 was created with a 1-cm margin to the GTV. All volumes were defined again on integrated PET-CT images. Lungs (right and left separately), liver, heart, esophagus, and kidneys (right and left) were counted as organs at risk in each patient. We set the window and level for the PET images according to method previously described by Erdi et al for accurate target volume definition [20]. In this protocol, we first measured the value of the hottest pixel in the lesion and then set the upper window level to this maximum value and set the lower window level to 42% of the maximum level.

### Statistical Analysis

On the basis of the literature, we hypothesized that integration of PET into RTP would change the target volumes in approximately 30% of the patients. To detect such a change with a 95% confidence interval of 5% to 55%, we needed to enroll at least 13 patients. Statistical differences between paired parameters from CT-based versus PET-CT-based treatment plans were evaluated with the Wilcoxon signed rank test. Results are expressed as mean ± standard deviation (SD). Differences were considered statistically significant when the two-tailed *P *value was less than .05.

## Results

Demographic and clinical characteristics of the 13 patients are depicted in Table 1. Four of the 13 patients were women. Median age was 50 years, with range of 38 to 74 years. In all but one patient, compared with CT-based delineation, PET-CT-based delineation resulted in significantly decreased mean GTV, CTV, PTV1, and PTV2 (Table 2). In these 12 patients, mean GTV decreased by 47.1% ± 28.4%, mean CTV decreased by 38.7% ± 24.7%, mean PTV1 decreased by 31.1% ± 23.1%, and mean PTV2 decreased by 40.0% ± 24.0%. In all 12 patients the respective target volume reductions were solely due to reduced primary tumor volumes on PET-CT fusion compared to CT with no change in nodal disease exclusion by PET data. In one patient, volumes were increased by PET-CT compared with CT; these increases were 19%, 2%, 10% and 15% in GTV, CTV, PTV1 and PTV2, respectively. This increament was due to additional involved lymph node detection by PET data which was not appearent on CT.

**Table 1:** Patient characteristics.

**Patient #**	**Sex**	**Age, y**	**ECOG**	**Stage**	**SUV_max_**	**SUVmean**
**1**	Male	50	1	T4N3M0	11.5	7.5
**2**	Female	54	1	T3N1M0	21.8	12.5
**3**	Male	74	0	T1aN3M0	10.9	5.1
**4**	Male	38	1	T4N1M0	4.2	2.3
**5**	Male	43	0	T1aN2 M0	6.7	3.5
**6**	Male	47	0	T2N0M0	6.5	4.1
**7**	Male	42	0	T1aN3M0	7.2	6.9
**8**	Female	71	1	T1aN3M0	9.8	5.6
**9**	Male	46	0	T3N0M0	13.2	8.6
**10**	Female	64	1	T1aN2M0	7.9	4.7
**11**	Male	74	0	T1aN2M0	15.3	8.4
**12**	Male	47	1	T4N2M0	12.0	6.9
**13**	Female	50	0	T3N1M0	9.5	6.8

*Abbreviations: ECOG, Eastern Cooperative Oncology Group; M, distant metastasis; N, lymph-nodal disease; T, tumor extension.

Stage was determined using the International Mesothelioma Interest Group criteria.

**Table 2:** Volumes by CT and PET-CT in 13 Patients with Malignant Pleural Mesothelioma.

**Volume, cc**	**CT**	**PET-CT**	***P *Value**
**GTV**			
Mean ± S.D.	788.9 ± 845.1	441.4 ± 420.0	0.01
Min-max	(101.4 - 3352.1)	(38.5 - 1250.2)	
**CTV**			
Mean ± S.D.	2040.6 ± 1360.5	1533.1 ± 1483.6	0.002
Min-max	(479.6 - 5615.8)	(254.4 - 5615.82)	
**PTV1**			
Mean ± S.D.	3824.7 ± 1777.7	2936.7 ± 1940.1	0.003
Min-max	(1062.5 - 7523.8)	(608.9 - 6971.9)	
**PTV2**			
Mean ± S.D.	2385.5 ± 1449.9	1627.9 ± 1254.2	0.003
Min-max	(488.2 - 5853.1)	(278.9 - 4088.4)	

*Abbreviations: CT = computed tomography; CTV = clinical target volume; GTV = gross tumor volume; PET-CT = positron emission tomography-computed tomography; PTV1 = planning target volume 1 (defined as CTV plus a 1-cm margin); PTV2 = planning target volume 2 (defined as GTV plus a 1-cm margin).

**Figure 1:**
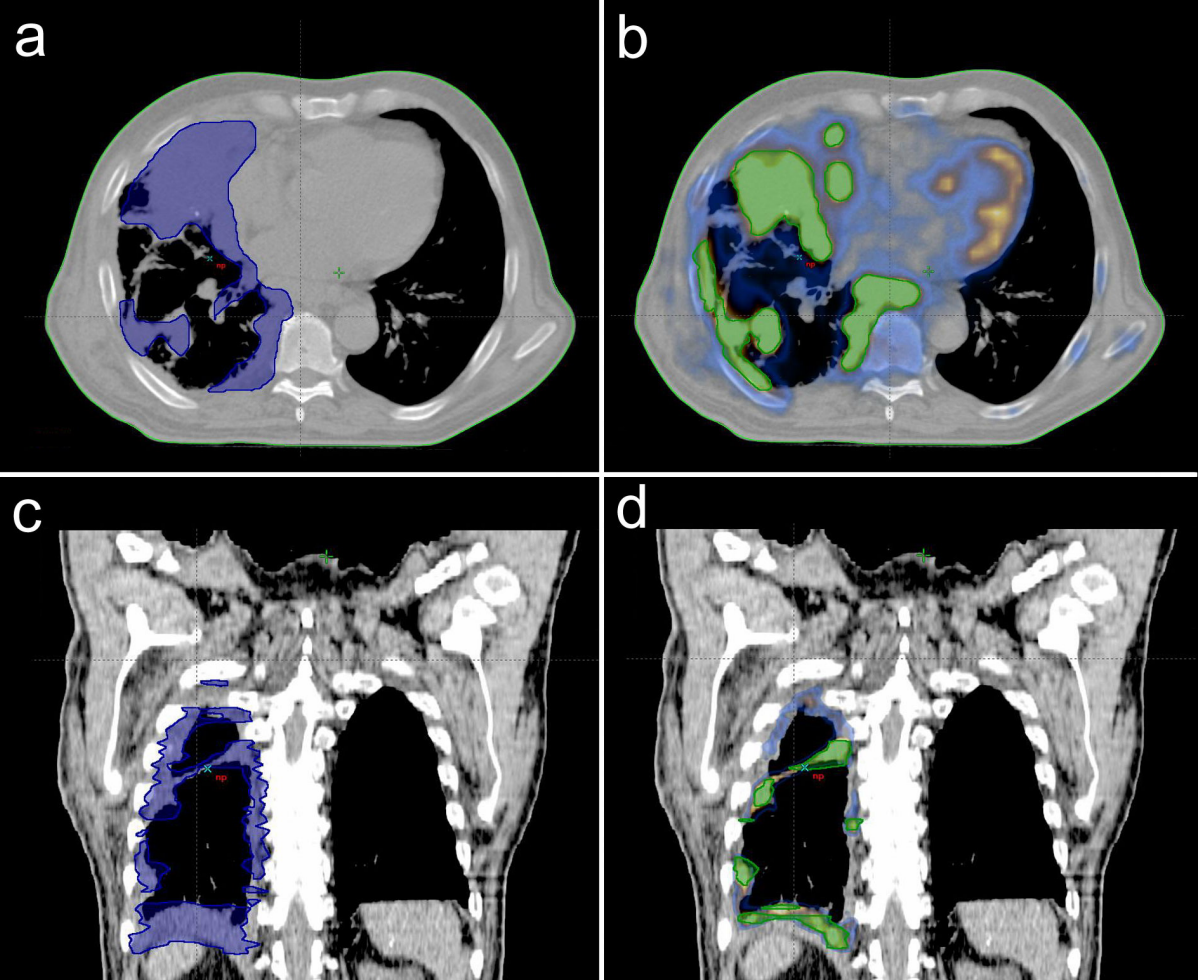
**Representative image of a patient with CT- and PET- CT based GTV delineations; (a) axial CT (b) axial PET-CT, (c) coronal CT, (d) coronal PET-CT**. *Abbreviations: GTV = gross tumor volume; CT = computed tomography; PET-CT = positron emission tomography-computed tomography.

In 4 of 13 patients (31%), PET-CT identified increased 18-fluorodeoxyglucose uptake in hilar lymph nodes that did not appear on CT, thereby changing the N stage in those 4 patients. In 3 patients (23%), PET-CT showed subdiaphragmatic extension of the disease which did not appear on CT. Representative images of a patient with different GTV delineations are seen in the Figure 1 and Figure 2.

## Discussion

On background of a nonexistent radiotherapeutic consensus for unresected nonmetastatic MPM in the literature, we performed a pure delineation study to evaluate the differences via implementation of PET-CT in order to generate potential possibilities for future radiotherapy decisions with current and coming cutting edge technologic advances. The results of the current study revealed that compared to CT, integrated PET-CT-based target volume delineation significantly reduced the GTV and its expansions, CTV and PTV, in 12 of 13 patients and increased target volumes in 1 patient, all together impacting the importance of accurate target volume delineation in this patients group. Additionally, we found that functional PET data changed the N stage in 4 of 13 patients, and subdiaphragmatic tumor extension was evident in further 3 (23%) patients that was not shown by CT, which may explain the possibility of geographic misses experienced with CT-based RTP and its influence on poor outcomes in patients with MPM.

There is currently no universally accepted standard therapy for MPM. Regarding the difficulties in diagnosis, staging, and treatment, it presents a unique therapeutic challenge. Currently, EPP with en bloc resection of the lung, pleura, ipsilateral diaphragm, and pericardium is the treatment of choice. However, only 10% to 15% of patients are eligible for this extensive surgery [5,6], and significant procedure-related morbidity and mortality limit its use. In addition, even with EPP, R0 resection is theoretically impossible, and microscopic or macroscopic disease almost always remains at the resection margins. When EPP is the sole treatment modality, locoregional recurrence is unacceptably high, ranging from 31% to 64% [21-23]. Therefore, postoperative RT is usually indicated. In a number of studies, high-dose hemithoracic RT of 45 to 50 Gy with a boost to 54 to 60 Gy targeted to areas at higher risk for local recurrence significantly improved local control [24-27]. In a study by Perrot et al, only 10% of patients developed recurrence in the ipsilateral hemithorax after completion of intended 60 Gy RT [24]. Similarly, Rusch et al demonstrated that adjuvant hemithoracic RT of 54 Gy following EPP improved local control with a 13% risk of local failure [26]. However, as was the case in our current cohort, the majority of patients with malignant pleural mesothelioma are not good candidates for curative EPP due to presence of either advanced local disease or unfavorable medical conditions that render them unfit for surgery.

In the setting of unresectable or medically inoperable/patient refusal conditions, RT when applied with pallative intent may offer good symptom control in conventional palliative doses. However, there is strong evidence suggesting better symptom and possibly loco-regional tumor control with higher doses approaching to that is used for curative intent. In one study, Ball et al showed that only 1 (4%) of 23 patients who received < 40 Gy achieved symptomatic relief while 4 (66%) of 6 patients treated with > 40 Gy had satisfactory symptom palliation impacting the importance of total dose even for palliative purposes [28]. Largely based on this data we planned to reassess our patients those who were treated with an palliative approach whether they were suitable for higher RT doses in the range of curative 54 Gy, as these patients theoratically still bear a chance for cure with higher RT doses even in absence of EPP. However, absence of a HT unit or a similar volumetric arc technology in our clinics significantly limited our ability to create clinically relevant and acceptable RTPs based on compatible pulmonary toxicity criteria. Therefore we planned to only compare the conventional CT- and PET-CT based target volume delineations which may positively impact and alter the future RTPs either for curatively or palliatively intended approaches in presence of HT facilities.

Despite the evident advantages offered by escalated doses with use of 3D- conformal RT it is not usually possible to escalate RT dose because of significant toxicity concerns Linden et al treated 47 MPM patients with a dose of 40 Gy in 20 fractions with and without chemotherapy and they informed that all of patients experienced radiation induced pulmonary fibrosis [29]. However, in this setting, more sophisticated RT techniques such as IMRT, IGRT, and especially helical-slit IMRT (HT) and cone-beam IMRT (RapidArc and VMAT) [30] might become appropriate alternatives for either definitive or palliative treatment for suitable patients based on compatible pulmonary toxicity criteria. Helical tomotherapy is a promising method, and achieves a better dose conformity in several tumor sites including MPM [31-33]. In their recent study, Sterzing et al compared step-and-shoot IMRT with HT [33]. They observed that while both modalities achieved excellent dose distributions, target coverage and homogenity could be increased significantly with HT, additionally contralateral lung dose could be lowered beyond 5 Gy. They concluded that HT is an excellent option for the IMRT of MPM. In our current study, as aforementioned it was impossible to create appropriate RTPs and guiding DVHs in absence of further technical advances such as HT oppurtunities, yet, we believe that the significant reductions observed in target volumes, additional subdiaphragmatic extension and involved lymph nodes shown only by PET data might be accepted as a useful evidence for future studies with appropriate technologies.

Although CT is the primary imaging modality for both staging and RTP in patients with MPM, the results of CT fail to identify the true extent of local invasion through the extrathoracic fascia, diaphragmatic surfaces, and interlobar fissures. Results of CT cannot accurately distinguish between malignant and benign conditions, such as inflammation and pleural fluid, which are common findings of MPM. Webb et al showed that the desmoplastic reaction caused by tumor-induced proliferation of benign connective tissue adjacent to the tumor can result in an overestimation of the stage of the tumor [34]. Additionally, CT has poor sensitivity for defining the malignant status of mediastinal lymph nodes [8,9]. Earlier studies in lung cancer showed significant changes when PET information was applied, with decreased volumes mostly attributed to exclusion of atelectasis [15,35-37]. We found that 18-fluorodeoxyglucose PET led to better definition of target volumes with additional metabolic information, and it was more successful in discriminating between tumor and benign connective tissue changes.

The additional volume and intratumoral functional variations uniquely identified by PET may be even more important in the near future when so-called dose-painting intensity-modulated radiotherapy becomes widely used in clinical practice, opening the possibility of controlled and reproducible internal-dose escalation to functionally important areas of the tumor. With the use of more specific functional PET tracers, this high-precision RT technique could help enormously in resolving the problems of overestimation and underestimation of GTV and mitigate their negative consequences for radiation management of tumors at many sites, including MPM.

We believe that our current study might be a significant contribution to the emerging RT literature regarding the use of PET-CT data in conjunction with CT in RTP of MPM patients. However, it is not appropriate to draw strict conclusions based on the current results without conformation of its use with novel sophisticated RT techniques such as HT with dose- volume histogram data which can predict RT related toxicity after curative or palliative RT. Therefore the present study seems to be a baseline data for further clinical and dosimetric studies rather than being considered as a guide.

## Conclusion

This study demonstrated the usefulness of PET/CT based target volume delineation in patients with MPM. The largest potential benefit of incorporating PET into RTP for MPM may be the reduction in geographic misses associated with CT-based planning, and, as a result, the potential reduction in local and regional treatment failures. However, we believe that before reaching more definite conclusions, more clinical studies are required to better define the role of PET-CT fusion in this setting.

## Competing interests

We have no personal or financial conflict of interest and have not entered into any agreement that could interfere with our access to the data on the research, or upon our ability to analyze the data independently, to prepare manuscripts, and to publish them.

## Authors' contributions

All authors read and approved the final manuscript. BP and ET carried out all CT evaluations, study design, target delineations, interpretation of the study, and drafted the manuscript. GNN carried out all PET evaluations and delineation of target volumes based on PET findings. CO carried out statistical analysis. OZ participated in manuscript preparation and study design. YD made the treatment planning. MNY, AAY gave advice on the work and helped in the interpretation of the data.
